# Clinicopathologic and prognostic significance of human epidermal growth factor receptor in patients with gastric cancer: An updated meta-analysis

**DOI:** 10.18632/oncotarget.15231

**Published:** 2017-02-09

**Authors:** Zhiqiao Zhang, Hongfeng Tang, Jixin Lin, Yunzhao Hu, Guanying Luo, Zhaowen Luo, Canchang Cheng, Peng Wang

**Affiliations:** ^1^ Department of Infectious Disease, The First People’s Hospital of Shunde, Shunde, Guangdong, China; ^2^ Department of Science and Education, The First People’s Hospital of Shunde, Shunde, Guangdong, China; ^3^ Department of Internal Medicine, The Chencun Hospital Affiliated to The First People’s Hospital of Shunde, Shunde, Guangdong, China

**Keywords:** Gastric cancer GC, human epidermal growth factor receptor, meta-analysis, prognostic significance

## Abstract

**Purpose:**

The aim of this update meta-analysis was to clarify the clinicopathologic and prognostic significance of human epidermal growth factor receptor(EGFR) expression in gastric cancer patients.

**Experimental Design:**

Several electronic databases were searched from January 1970 to May 2016. The odds ratio (OR) was calculated to assess the association between EGFR expression and pathological parameters. The hazard ratio (HR) and 95% CI were calculated to explore the relationship between EGFR expression and overall survival.

**Results:**

Finally 7229 patients with gastric cancer from 25 eligible studies were included in the present meta analysis. High EGFR expression was found to be significantly related with tumor differentiation (OR=1.96, 95%CI: 1.14-3.34, *Z*=2.43, *P*=0.015), lymph node metastasis (OR=2.20, 95% CI: 1.63-2.96, *Z*=5.17, *P*=0.001), and tumor stage (OR=2.13, 95% CI: 1.35-3.36, *Z*=3.25, *P*=0.001). However, high EGFR expression was not significantly associated with invasion depth (OR=2.09, 95% CI: 0.4-11.05, *Z*=0.87, *P*=0.385). The pooled HR suggested that high EGFR expression was significantly correlated with overall survival (HR=1.19, 95% CI 1.04-1.37, *Z*=2.44, *P*=0.015).

**Conclusions:**

The present meta-analysis demonstrated that high EGFR expression significantly predicts poor prognosis, suggesting that high EGFR expression may serve as a predictive biomarker for poor prognosis in patients with gastric cancer.

## INTRODUCTION

It has been reported that the fourth most malignant tumor is Gastric cancer (GC), which is the second leading cause of tumor related death in the world [1]. It was estimated that there were 24,590 new patients diagnosed with GC in the United States with 10,720 deaths in 2015 [2]. Patients with advanced GC have a median overall survival of less than 12 months [3–6]. From the aspect of active treatments and preventions, reliable prognostic biomarkers are valuable for improvement of prognosis in GC patients.

The human epidermal growth factor receptor (EGFR) is a cell membrane tyrosine kinase receptor. The prognostic significance of EGFR expression in GC patients was controversial in different studies. High EGFR expression has been reported to be correlated with poor prognosis in patients with GC [7–8]. However, it has been reported that patients with high EGFR expression had favorable prognosis than those with low EGFR expression [9]. Furthermore, EGFR expression has been reported to be not significantly associated with overall survival in patients with GC [10]. Even more interesting, the conclusions in two meta analyses were controversial too. Hong et al. reported that high EGFR expression was not an independent predictor for prognosis of GC patients [11]. However, Chen et al. reported that high EGFR expression had a significant predictive ability for prognosis in GC patients and might be useful for predicting prognosis of GC patients [12].

These opposed conclusions leaded to great confusion about prognostic significance of EGFR expression in patients with GC. Therefore, we performed this update meta-analysis to determine the clinicopathologic and prognostic significance of EGFR expression in GC patients.

## RESULTS

### Search results

**Figure 1:**
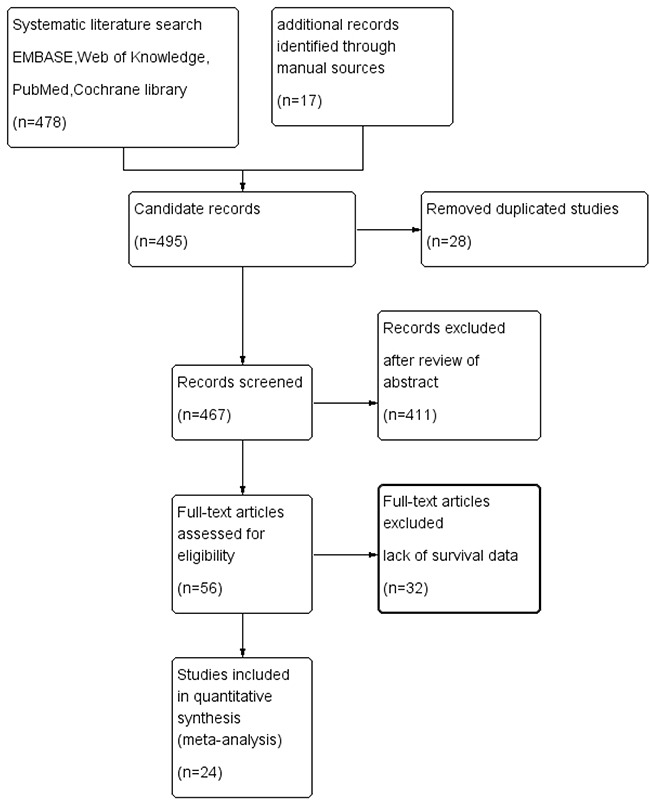
Flowchart of study selection in the present meta-analysis

The initial search returned 495 studies (with 28 duplicate studies). After screening the abstracts, 411 irrelevant studies were excluded according to the criteria of inclusion and exclusion. Reviewers identified 56 potential studies for full-text review and 31 studies were eliminated due to inadequate data for meta-analysis. Finally, 25 studies were included in the current meta-analysis [7, 9, 10, 19–40]. The detail of the screening process was shown in Figure 1.

### Study selection and characteristics

The characteristic of the included studies were summarized in Table 1. The publication time ranged from 1993 to 2016. The subject number of the included studies ranged from 30 to 950, with a mean sample size of 289. The mean length of follow-up period ranged from 11 to 96 months. The NOS score of 25 eligible studies varied from 7 to 8, with a mean value of 7.16. Twenty-five studies provided overall survival data and/or survival curves. Fifteen studies explored the association between EGFR expression and clinicopathologic parameters, such as invasion depth, tumor differentiation, tumor stage, and lymph node metastasis.

**Table 1:** Characteristics of studies included in the present meta analysis

Author	Country	Method		Number	Mean	Male			HR		
Year	Language	Cut-off value	Studytime	Positiverate	Age range	Female	Tumorstage	Follow-upperiod	95%CI	*P* value	NOS score
Hironoet al 1995	Japan English	IHC≥1+	1983-1990	10339.8%	NR	NR	stage II–IV	NR	1.15(0.65-2.06)	0.631	6
Jonjicet al 1997	Italy English	IHC≥1+	1987-1989	5653.6%	70(40-87)	31/25	stage I–IV	NR	2.33(1.25-4.36)	0.008	7
Songet al 2004	Korea English	IHC≥10%	1996-2001	73925.4%	59(19-80)	495/244	stage I–III	31(1-97)	0.99(0.56-1.73)	0.972	8
Langeret al 2006	Germany English	IHC≥10%	1991-2002	13755%	63(33-83)	125/12	stage I–III	36	1.01(1-1.02)	0.039	7
Galiziaet al 2007	Italy English	IHC≥1%	1996-2005	8244%	62(34-83)	51/31	stage I–IV	49(6-12)	2.97(1.22-7.22)	0.017	7
Matsubaraet al 2008	Japan English	IHC≥10%	1997-2004	8763%	64	70/17	un-resectable or recurrent	NR	0.99(0.63-1.57)	0.97	7
Kimet al 2008	Korea English	IHC≥2+	1999	51127.4%	55.4	NR	stage I–IV	68(1-108)	1.84(1.35-2.49)	0.001	7
Kimet al 2009	Korea English	IHC≥1+	1995-2003	15380.7%	52(15-72)	108/45	stage III–IV	72.9(2-135)	0.605(0.37-0.99)	0.045	8
Czyzewskaet al 2009	Poland English	IHC≥50%	1996-1998	5554.5%	60(30-78)	17/38	stage I–IV	84	1.09(0.53-2.25)	0.815	7
Inokuchiet al 2011	Japan English	IHC≥10%	1999-2002	12629%	NR	88/38	stage I–IV	73(2-135)	2.2(0.99-4.9)	0.053	7
Zhanget al 2011	China Chinese	IHC≥1+	2001-2008	8455.9%	55(22-84)	47/37	stage II–IV	11	1.33(0.71-2.5)	0.37	7
Atmacaet al 2012	Germany English	IHC≥1+	NR	35757.4%	NR	214/143	stage IV	18.2(3.3-44.1)	0.91(0.66-1.16)	0.464	8
Terashimaet al 2012	Japan English	IHC≥3+	NR	8299%	NR	565/264	stage II–III	60	1.64(1.14-2.37)	0.008	7
Al-Moundhri et al 2012	Oman English	IHC≥10%	1995-2005	11513.9%	59.2(21-90)	72/43	stage I–IV	96	1.72(1.09-2.7)	0.02	7
Gaoet al 2013	China English	IHC≥50%	2000-2007	7857.7%	NR	40/38	stage I–IV	NR	2.07(0.88-4.87)	0.096	7
Liet al 2013	China Chinese	IHC≥2+	2006	16146%	61(33-80)	124/37	stage I–IV	39.6	1.01(0.55-1.85)	0.974	7
Kandelet al 2013	France English	IHC≥2+	1999-2002	8216.3%	67(38-95)	58/24	stage I–III	40	1.68(0.82-3.46)	0.158	7
Aydinet al 2013	Turkey English	IHC≥2+	2008-2011	3063.3%	34-85	20/10	stage II–IV	12(2-25)	0.36(0.1-1.23)	0.118	7
Kurokawaet al 2014	Japan English	IHC≥10%	2000-2006	15314.4%	68(35-98)	104/49	stage I–IV	NR	1.78(0.94-3.38)	0.077	8
Fuseet al 2014	Japan English	IHC≥2+	2006-2010	29327%	≥20Y	201/92	unresectable or recurrent	58.4	1.12(0.86-1.46)	0.401	7
Tanget al 2014	China English	IHC≥2+	2007-2010	12133.1%	NR	85/36	stage II–IV	NR	1.41(0.73-2.74)	0.306	7
Nagatsumaet al 2014	Japan English	IHC≥2+	2003-2007	95023.5%	63(18-92)	734/316	stage I–IV	60(1-120)	0.58(0.39-0.87)	0.007	7
Paligaet al 2015	Canada English	IHC≥2+	2002-2008	11315%	64(30-94)	81/32	stage I–IV	80(73-93)	1.6(0.89-2.87)	0.11	8
Seoet al 2015	Korea English	HC≥2+	2003-2010	87912.6%	NR	NR	stage I–IV	NR	0.66(0.27-1.65)	0.377	7
Parket al 2016	Korea English	IHC≥3+	2000-2003	93514.7%	59(25-86)	618/317	stage I–III	NR	0.92(0.69-1.22)	0.57	7

* NOS, Newcastle-Ottawa Quality Assessment Scale; HR, hazard ratio; CI, confidence interval.

### Association of EGFR expression with clinicopathologic parameters

**Figure 2:**
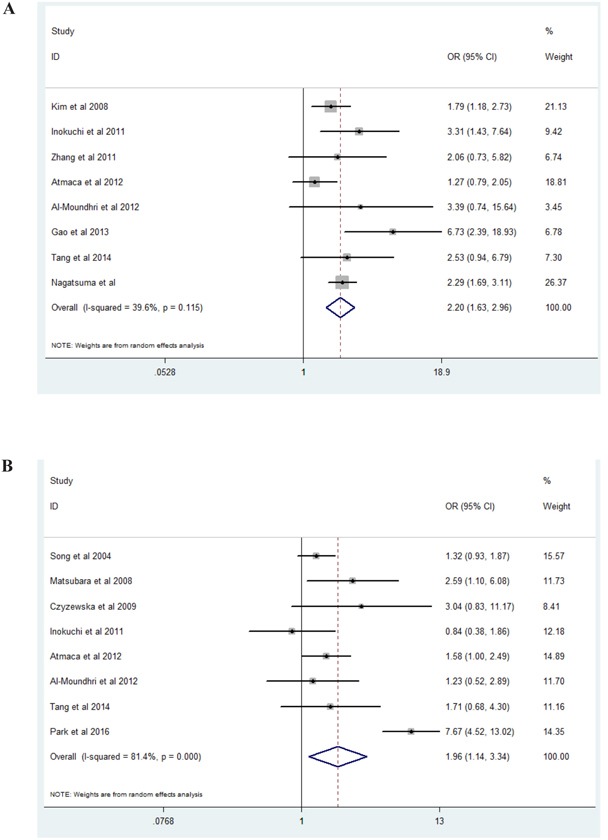

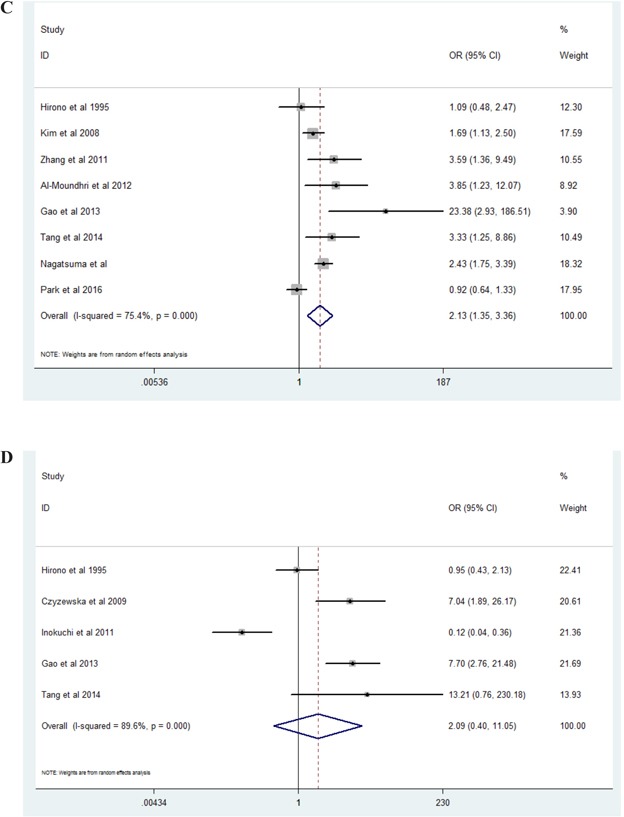
Forest plots of studies evaluating the correlation between EGFR expression and pathological parameters **A**. Lymph node metastasis (present vs absent); **B**. Tumor differentiation (poor vs well/moderate); **C**. Tumor stage (I-II vs III-IV); **D**. Invasion depth (present serosal invasion vs absent serosal invasion).

The correlations between high EGFR expression and clinicopathologic parameters were presented in Figure 2. The random effects model was used for significant heterogeneity. As shown in Figure 2, high EGFR expression was found to be significantly related with lymph node metastasis (Figure 2A, present vs absent, OR=2.20, 95% CI: 1.63-2.96, Z=5.17, P=0.001), tumor differentiation (Figure 2B, poor vs well/moderate, OR=1.96, 95%CI: 1.14-3.34, Z=2.43, P=0.015), and tumor stage (Figure 2C, I-II vs III-IV, OR=2.13, 95% CI: 1.35-3.36, Z=3.25, P=0.001). However, high EGFR expression was not significantly associated with invasion depth (Figure 2D, present serosal invasion vs absent serosal invasion, OR=2.09,95% CI: 0.4-11.05, Z=0.87, P=0.385).

### Prognostic significance of EGFR expression in gastric cancer patients

**Figure 3:**
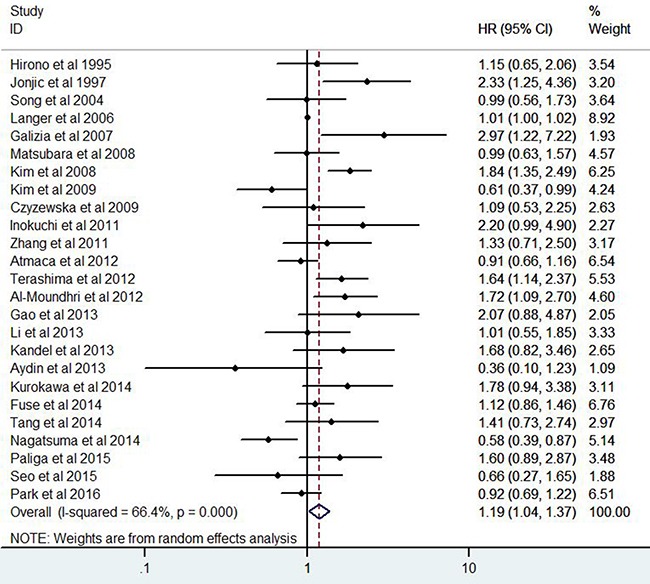
Forest plots of studies evaluating the hazard ratio of EGFR expression for overall survival

A total of 7229 patients with GC from 25 eligible studies were collected and analyzed (Figure 3). The pooled HR was 1.19(95% CI 1.04-1.37, *Z*=2.44, *P*=0.015). The results demonstrated that high expression of EGFR significantly was related with poor prognosis for patients with GC.

### Sensitivity analysis

All studies were sequentially removed to explore that whether any individual study had a significant influence to the pooled HR. The results of sensitivity analysis demonstrated that the pooled HR was not significantly affected by any study (Table 2).

**Table 2:** Effect of individual studies on the pooled HRs of EGFR expression and overall survival

Study omitted	HR	Lower value of 95% CI	Upper value of 95% CI
Hirono et al 1995	1.1928611	1.0328844	1.3776154
Jonjic et al 1997	1.1617202	1.0121113	1.3334442
Song et al 2004	1.1998487	1.0386479	1.3860684
Langer et al 2006	1.2199296	1.0275393	1.4483418
Galizia et al 2007	1.1671815	1.0171522	1.3393401
Matsubara et al 2008	1.2024842	1.0395564	1.3909475
Kim et al 2008	1.1484013	1.0027492	1.3152098
Kim et al 2009	1.2254386	1.063726	1.4117356
Czyzewska et al 2009	1.1940148	1.0348687	1.3776351
Inokuchi et al 2011	1.1723867	1.0195748	1.3481018
Zhang et al 2011	1.1865808	1.0283683	1.3691339
Atmaca et al 2012	1.2157478	1.0466765	1.4121294
Terashima et al 2012	1.1663924	1.0124791	1.3437031
Al-Moundhri et al 2012	1.1679779	1.0142503	1.3450057
Gao et al 2013	1.1759335	1.0220827	1.3529429
Li et al 2013	1.1981824	1.0376033	1.3836125
Kandel et al 2013	1.1790264	1.0234496	1.3582527
Aydin et al 2013	1.2049856	1.0482415	1.3851677
Kurokawa et al 2014	1.1744384	1.0198769	1.3524236
Fuse et al 2014	1.1992651	1.0315969	1.3941848
Tang et al 2014	1.1845405	1.0270487	1.3661827
Nagatsuma et al 2014	1.2351219	1.0736278	1.4209078
Paliga et al 2015	1.177506	1.0215301	1.3572975
Seo et al 2015	1.2041327	1.045177	1.387263
Park et al 2016	1.2147179	1.0458301	1.4108788
combined	1.1901691	1.0350099	1.3685883

### Publication bias

**Figure 4:**
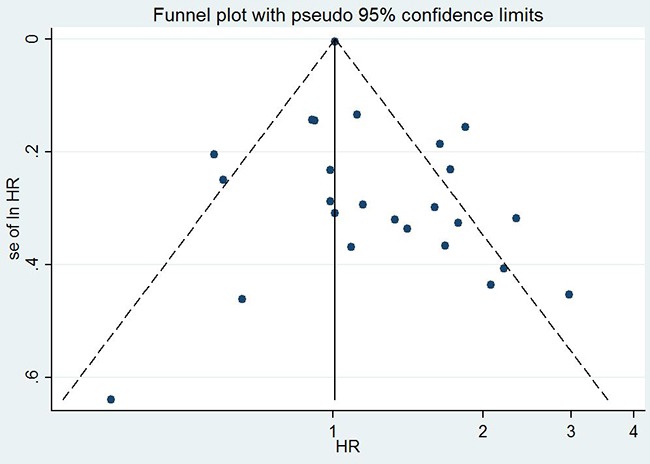
The funnel plot for all eligible studies provided HRs of EGFR expression for overall survival

The funnel plot (Figure 4) was significantly asymmetry for overall survival. The Begg’s test did not find significant evidences for publication bias in terms of invasion depth(*P*=1.0), tumor stage(*P*=0.266), tumor differentiation(*P*=0.386), lymph node metastasis(*P*=0.711), and overall survival (*P*=0.266). The Egger’s test indicated that there were no evidences for publication bias in terms of invasion depth (*P*=0.613), tumor stage(*P*=0.192), tumor differentiation(*P*=0.803), and lymph node metastasis(*P*=0.216). However, the Egger’s test demonstrated that there might be potential publication bias (*P*=0.051) for overall survival.

### Explore the causes of the funnel plot asymmetry by Contour-enhanced funnel plot

**Figure 5:**
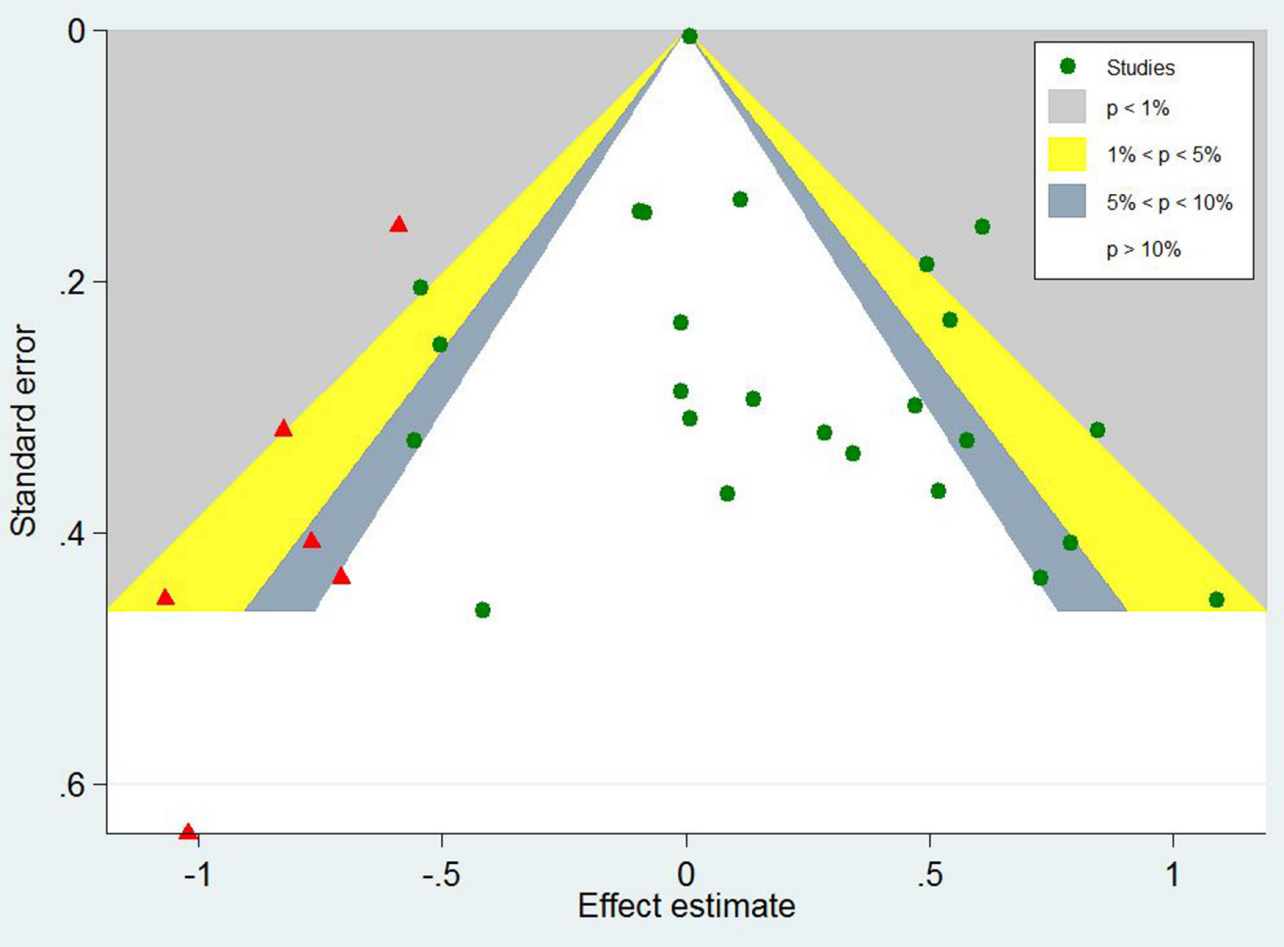
Contour-enhanced funnel plot with trim-and-fill method for OS in GC patients

The contour-enhanced funnel plot with the trim-and-fill method can help to determine whether or not the funnel plot asymmetry was caused by publication bias [18]. Contour lines which suggested conventional milestones in levels of statistical significance (0.01, 0.05, and 0.1) are added to conventional funnel plots. If the dummy studies lie in the areas with high statistical significance (*P*>0.05), this will suggest that the funnel plot asymmetry may be caused by factors other than publication bias, such as poor methodological quality or true heterogeneity. If the dummy studies lie in the areas with non-statistical significance, then this will indicate the possibility that the funnel plot asymmetry is due to publication bias. The dummy studies were indicated by red triangles and the genuine studies were indicated by green dots (Figure 5).

The contour-enhanced funnel plot with trim-and-fill method finally added 6 dummy studies to balance the funnel plot and 3 dummy studies lied in the areas with high statistical significance, indicating that the publication bias was not the only cause for the funnel plot asymmetry.

### Stability assessment of EGFR expression for prognosis by cumulative meta-analysis method

**Figure 6:**
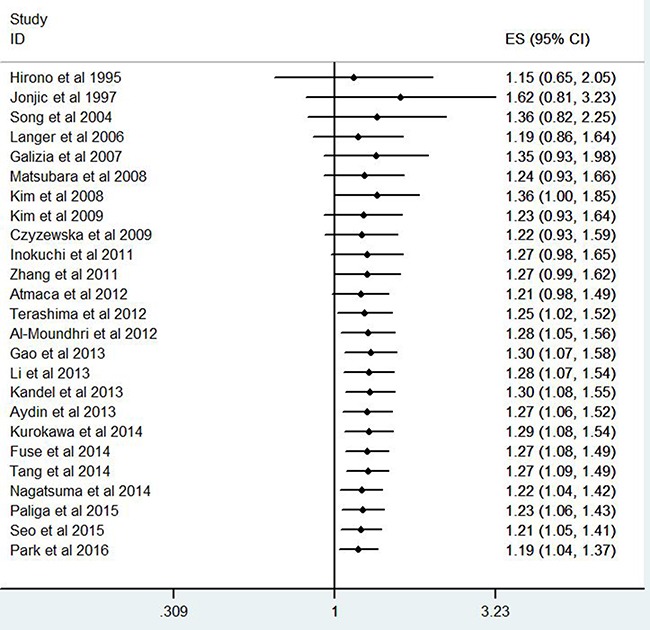
Cumulative meta-analysis for stability of EGFR expression for prognosis of GC patients

We further performed cumulative meta-analysis to assess the stability of EGFR expression for prognosis of GC patients (Figure 6). The pooled HRs of cumulative meta-analysis ranged from 1.19(95%CI: 1.04-1.37) to 1.30 (95%CI: 1.08-1.55) for OS since 2012, demonstrating that performance of EGFR expression for prognosis in GC patients was stable and reliable.

### Explore of sources of heterogeneity by meta-regression analysis and subgroup analysis

Meta-regression analysis and subgroup analysis were performed to explore the sources of heterogeneity. However, we did not find any significant source of heterogeneity using meta-regression analysis (data not shown) and subgroup analysis (Table 3).

**Table 3:** Subgroup analysis for association between EGFR expression and overall survival in GC patients

		Overall survival		95%CI		Heterogeneity	
Group factors	Subgroup	HR	*P* value	Lower	Upper	*I*^ 2^	*P* value
Total	Total	1.19	0.015	1.04	1.37	66.4%	0.001
Contain stage IV patients	Yes	1.11	0.302	0.91	1.37	55.9%	0.059
	No	1.22	0.052	1.0	1.5	65.4%	0.001
Chemistry therapy	Yes	1.21	0.291	0.85	1.71	65.4%	0.008
	No	1.18	0.042	1.01	1.38	65.8%	0.001
Patients number≥100	Yes	1.13	0.113	0.97	1.31	68.5%	0.001
	No	1.44	0.044	1.01	2.05	48.3%	0.06
Positive rate≥50%	Yes	1.04	0.744	0.84	1.28	54.7%	0.024
	No	1.29	0.012	1.06	1.59	63.1%	0.001

HR, hazard ratio; CI, confidence interval.

Subsequently all studies were sequentially removed to explore that whether any individual study had a significant influence to the heterogeneity. The results showed that the study conducted by Kim et al. had the most significant impact in all 25 studies. The heterogeneity decreased from 66.4% to 59.4% after removing the study conducted by Kim et al.

## DISCUSSION

Through the present meta analysis, we explored the clinicopathologic and prognostic significance of EGFR expression in GC patients. The combined ORs indicated that high EGFR expression was significantly correlated with tumor differentiation, lymph node metastasis, and tumor stage. The pooled HRs demonstrated that high EGFR expression significantly predicted poor OS compared with low EGFR expression. The results of sensitivity analysis and cumulative meta-analysis demonstrated that the performance of EGFR expression for prognosis in GC patients was stable and reliable.

It has been reported that the activation of EGFR leads to the phosphorylation of the intracellular tyrosine kinase and initiate a series of intracellular signal pathways such as the STAT, Ras-MAPK, and PI3K-AKT signaling pathway [8–9]. It has been found that these signaling pathways above can affect cell proliferation, angiogenesis, invasion, migration, metastasis, and apoptosis [41–42]. These studies provided reasonable interpretation of molecular biology for the prognostic role of EGFR expression in patients with GC.

The conclusion of the present meta analysis was different to that of two previous meta analyses. The differences in three different meta analyses might be caused by the following reasons. Firstly, the patient number in the present meta analysis was 7229, which was significantly more than that of two previous meta analyses (1289 and 1600, respectively). Secondly, the number of eligible studies included in two previous meta analyses were comparatively small (only five studies and seven studies, respectively), which might reduce the convincingness of the conclusions. Thirdly, selection bias might exist in these previous meta analyses for the reason that some eligible studies did not include in these two meta analyses. Fourthly, some studies with EGFR expression detected by Polymerase Chain Reaction (PCR) were included in meta analysis performed by Chen et al. [12] and might lead to clinical heterogeneity. Fifthly, Jácome AA et al. explored the association between EGFR expression and overall survival by Weibull model method [43], which was significantly different to COX regression model and might lead to statistical heterogeneity. To reduce clinical heterogeneity, the present meta analysis did not include the study performed by Jácome AA.

We detected significant heterogeneity in the current meta analysis. There might be some potential sources of heterogeneity such as cut-off values, tumor stages, treatments, and races. However, we did not find any significant source of heterogeneity using meta-regression analysis and subgroup analysis.

Publication bias is an important factor for interpretation of the conclusions. The funnel plot was asymmetry and the Egger’s test suggested that the existence of potential publication bias. The contour-enhanced funnel plot by using the trim-and-fill method was performed to explore the potential sources for the funnel plot asymmetry. There were 3 studies out of 6 added dummy studies lying in the areas with high statistical significance, meaning that the publication bias might not the only cause to the funnel plot asymmetry. The funnel plot asymmetry might be caused by the following factors such as language bias, location bias, citation bias, multiple publication bias, true heterogeneity, poor methodological quality of little sample studies, and selective reporting.

Although significant heterogeneity and publication bias were detected in the present meta-analysis, further sensitivity analyses ascertained that the prognostic significance of high EGFR expression in GC patients did not changed after removing any study. Meanwhile, cumulative meta-analyses also supported that the performance of high EGFR expression for prognosis of GC patients was stable and reliable.

The present meta analysis have several strengths: First, we first revealed that high EGFR expression is significantly correlated with tumor differentiation, lymph node metastasis, and tumor stage. Second, we included 25 eligible studies and 7229 GC patients through comprehensively search in several electronic databases and additional manual search. Third, all detection methods of EGFR expression were IHC, leading to less clinical heterogeneity. Fourth, studies published in Chinese were also included in the current meta analysis as English literature, increasing representation of the study population.

There were several limitations must be taken into account while interpreting the conclusions of the present meta analysis. First, high EGFR expression was defined according to different cut-off values in various studies, which might affect the stability of the conclusions in different studies. Second, different baseline characteristics, such as tumor stages, treatments, and races, might lead to clinical heterogeneity. Third, to reduce clinical heterogeneity, the studies with EGFR expression detected by Western blot (WB) or Polymerase Chain Reaction (PCR) were excluded from the present study, which might influence the clinical applicability. We recommend that the studies with EGFR expression detected by WB or PCR method should be included in future study to further explore the prognostic role of EGFR expression in GC patients. Fourth, the present meta analysis only provided evidences for correlation between high EGFR and clinicopathologic features, which could not be simply interpreted as causal relationship.

In conclusion, the present meta analysis demonstrated that high EGFR expression is correlated with poor OS, tumor differentiation, lymph node metastasis and tumor stage. Therefore, EGFR expression may serve as a valuable biomarker for predicting tumor prognosis in GC patients.

## MATERIALS AND METHODS

### Search strategy

A systematic search was performed in the following electronic databases: PubMed, EMBASE, Cochrane Library, and Wed of Knowledge database from Jan 1970 to May 2016 for eligible studies, which assessing the clinicopathologic and prognostic significance of EGFR expression for prognosis in GC patients. We performed literature search by strategy combined text word and MeSH (Emtree for EMBASE database accordingly) with the terms “EGFR” or “ErbB1” or “HER1” or “epidermal growth factor receptor” and “gastric cancer” or “gastric carcinoma” or “stomach tumor” and “survival” or “outcome” or “prognosis” or “prognostic”. The strategy was correspondingly adjusted in different databases. In the retrieval process, expanded search of hyponym was performed. Additional, we made a manual search using the reference lists of the included studies for including eligible studies. We even contacted the corresponding author to get necessary information if necessary. The search was restricted to human studies, but there were no restrictions on language or publication time. All clinical investigation and data achievement were conducted according to the principles of Declaration of Helsinki.

### Criteria for inclusion and exclusion

The inclusion criteria were as follows: (1) proven pathological diagnosis of GC in humans; (2) EGFR expression evaluation using immunohistochemistry (IHC) method; (3) provided information on clinicopathological parameters and/or survival outcome such as hazard ratio (HR) and 95% confidence interval (CI). Studies not directly providing hazard ratio and 95% confidence interval were included if survival information were available from survival curves. Studies published in Chinese were included in the current meta-analysis as English literature. Only the most recent study was included in the current meta-analysis among duplicate studies. There were no restrictions on sample size or follow-up period.

The following studies were excluded: (1) reviews, letters, case reports, and conference abstracts without original data; (2) non-human experiments;(3) laboratory studies;(4) studies from which the necessary information could not be extracted.

### Quality assessment of studies

Two reviewers (Zhiqiao Zhang and Jixin Lin) independently assessed the quality of the studies included in the present meta-analysis using the Newcastle-Ottawa Quality Assessment Scale (NOS) (Table 1). The NOS contains assessments of patient selection, study comparability, follow-up, and outcome of interest. The total scores were used to compare study quality. Disagreements in the literature assessment were resolved through consensus with the third reviewer (Hongfeng Tang).

### Data extraction

Two investigators (Zhiqiao Zhang and Jixin Lin) independently extracted and examined the following data from the original studies: surname of the first author, publication year, country, sample size, disease stage, detection method of EGFR expression, clinical parameters, and survival outcome data (HR and CI). Information from each eligible study was extracted and recorded in a standardized form. All eligible studies were coded as surname of the first author + publish year in the standardized form. When it was necessary, study authors were contacted for necessary information. Disagreements between two investigators were resolved by discussion. When necessary, the third investigator (Hongfeng Tang) helped to reach a consensus.

### Statistical analysis

The statistical analysis was executed according to the proposals of the Meta-Analysis of Observational Studies in Epidemiology group (MOOSE) [13]. The HRs and 95% CIs were used to summary survival information. We directly obtained pooled HRs and 95% CIs if the survival data were reported in the text. While the HRs and 95% CIs were not directly reported in the text, the survival information were extracted from Kaplan-Meier curve and used to estimate HR. The heterogeneity was assessed by using *I*^2^ statistic, which was defined according to the Cochrane Handbook [14]: 0% to 40%, negligible heterogeneity; 30% to 60%, moderate heterogeneity; 50% to 90%, substantial heterogeneity; 75% to 100%, considerable heterogeneity. The subsequently meta-analysis was performed using random effect model with DerSimonian and Laird method [15], which applying the inverse of variance as a weighing factor. Meta-regression analyses with REstricted Maximum Likelihood (REML) method and subgroup analyses were performed to explore the sources of heterogeneity. Funnel plot, Begg's test [16], and Egger's test [17] were used to assess the publication bias. The contour-enhanced funnel plot with the trim-and-fill methodwas performed to determine whether or not funnel plot asymmetry was caused by publication bias [18]. *P* value<0.05 was considered statistically significant. The statistical analyses were performed by STATA version 12.0 software (Stata Corporation, College Station, Texas, USA).

The funders had no role in study design, data collection and analysis, decision to publish, or preparation of the manuscript.

## Abbreviations

GC: Gastric cancer
EGFR: human epidermal growth factor receptor
OR: odds ratio
HR: hazard ratio
CI: confidence interval
